# Nutrition and cognition across the lifetime: an overview on epigenetic mechanisms

**DOI:** 10.3934/Neuroscience.2021024

**Published:** 2021-07-15

**Authors:** Arianna Polverino, Pierpaolo Sorrentino, Matteo Pesoli, Laura Mandolesi

**Affiliations:** 1 Institute of Diagnosis and Treatment Hermitage Capodimonte, Naples, Italy; 2 Department of Motor and Wellness Sciences, University of Naples “Parthenope”, Naples, Italy; 3 Institut de Neurosciences des Systèmes, Aix-Marseille University, Marseille, France; 4 Institute of Applied Sciences and Intelligent Systems, National Research Council, Pozzuoli, Italy; 5 Department of Humanities Studies, University of Naples Federico II, Naples, Italy

**Keywords:** nutrition, cognitive functioning, environmental factors, epigenetics, neuroplasticity

## Abstract

The functioning of our brain depends on both genes and their interactions with environmental factors. The close link between genetics and environmental factors produces structural and functional cerebral changes early on in life. Understanding the weight of environmental factors in modulating neuroplasticity phenomena and cognitive functioning is relevant for potential interventions. Among these, nutrition plays a key role. In fact, the link between gut and brain (the gut-brain axis) is very close and begins *in utero*, since the Central Nervous System (CNS) and the Enteric Nervous System (ENS) originate from the same germ layer during the embryogenesis. Here, we investigate the epigenetic mechanisms induced by some nutrients on the cognitive functioning, which affect the cellular and molecular processes governing our cognitive functions. Furthermore, epigenetic phenomena can be positively affected by specific healthy nutrients from diet, with the possibility of preventing or modulating cognitive impairments. Specifically, we described the effects of several nutrients on diet-dependent epigenetic processes, in particular DNA methylation and histones post-translational modifications, and their potential role as therapeutic target, to describe how some forms of cognitive decline could be prevented or modulated from the early stages of life.

## 1. Introduction

Complex interactions between genetic and environmental factors are needed for the correct functioning of the human brain [1]. The brain continuously elaborates information coming from the environment. It has been demonstrated that experience shapes brain structure and function, determining neuroplasticity phenomena such as neurogenesis, synaptogenesis, and spinogenesis [2]–[4].

Given the great influence of environmental factors in modulating plasticity processes, many researchers have focused on how these factors affect cognition [5]. Although there is evidence showing how levels of education, lifestyle (i.e. level of stress, quality of sleep, smoking, etc), social relations (i.e. affections and friendships), and motor activity (i.e. physical exercise) induce positive effects on cognitive functioning [6],[7], a growing body of literature demonstrated that also eating habits (i.e. nutrient intake) induce structural and functional changes in the brain, affecting cognition [8]–[10].

In this context, a healthy diet counterbalances age-related cognitive decline. In fact, diet has a positive impact on cognitive functions, by modulating a wide range of biological mechanisms such as neuronal cell membrane fluidity, synaptic plasticity, neuroinflammation, oxidative stress, neuroprotection and neurogenesis. Furthermore, these processes are not limited to adulthood, bur start early on during the embryonic development [10].

To better understand the link between dietary nutrients and the brain, one needs to take into account that the interactions between the gut and the brain begin during gestation. In fact, fetal neurodevelopment is a complex process depending, among other things, on environmental factors and molecular signals coming from the maternal microbiome. The latter plays a role in neurogenerative processes, such as blood-brain barrier formation, myelination, neurogenesis and glia maturation [11]. The maternal intestinal microbiome can affect the neurodevelopment, the microbiome and the behavior of the offspring, both in animal models [12] and humans [13].

Among eating habits with long-term beneficial effects, the Mediterranean diet plays a dual role. On the one hand, the Mediterranean diet can reduce the risk of cardiovascular diseases, cancer, diabetes and metabolic disorders [14]–[16]. On the other hand, it improves cognitive functions by preventing neuropathological processes such as mild cognitive impairments (MCI) [17], counteracting impairment in motor, executive, attention, memory, language and visuospatial functions characterizing Parkinson's disease (PD) [18], and other forms of dementia [19]. In fact, such diet is rich in antioxidant molecules due to its content in fruit, vegetables and olive oil [20], and in anti-inflammatory nutrients such as n-3 polyunsaturated fatty acids (n-3 PUFAs), from fish and olive oil [21], and in fibers, contained in fruit and vegetables [22],[23].

Several nutrients from the Mediterranean diet play a role in neuroprotection and prevention of neuronal diseases. For example, it was already known that n-3 PUFAs have a neuroprotective role, since n-3 PUFAs-enriched diets counteract age-related neurodegeneration, increase hippocampal neurogenesis, neuronal and microglia cells density and dendritic arborization of neurons, and reduce apoptosis, astrocytosis and lipofuscin accumulation in both animal models and humans [9],[24]–[27]. This is because n-3 PUFAs are able to improve neurotransmission and cell signaling through growing neuronal cell membrane fluidity, increased receptor number and ionic channels functionality [28]–[30]. Docosahexanoic acid (DHA) is the most abundant among the n-3 PUFAs in the brain cell membranes [31]. For this reason, its intake through the consumption of fish has been fundamental for encephalization during the human evolution [32]. Furthermore, n-3 PUFAs deficiency leads to impaired learning and memory in rodents [33],[34], while it increases the risk of several mental disorders in humans, such as dementia, depression, bipolar disorder and schizophrenia [35]–[37].

Polyphenols, such as curcumin, resveratrol and flavonoids also play an anti-inflammatory and neuroprotective role [38],[39], improving cognitive functions [40],[41] such as spatial working memory, in association with an increased expression of the Brain-Derived Neurotrophic Factor (BDNF) in animal models [42]. Furthermore, they are associated with better evolution of the cognitive performance over time in the elderly [43].

Vitamins B also play an important role in improving cognitive functions in both animal models and human. For example, vitamin B12 counteracts cognitive impairment in rats fed a choline-deficient diet [44], while it was demonstrated that vitamins B6, B12 and folate have positive effects on memory performance in women of different ages [45]. Vitamins B deficiency, and the resulting hyper-homocysteinemia, may affect cognition with loss of total brain volume and cognitive and memory decline [46],[47], through pathways involving redox potentials with altered calcium influx, tau protein and beta-amyloid accumulation, which are associated with AD pathogenesis [48]–[50], apoptosis and neuronal death [51].

Together with vitamins B, vitamins D and E play an important role in preserving cognition in the elderly, counteracting cognitive impairment [52]–[54]. In fact, a severe vitamin D deficiency significantly increases the risk of developing AD and other forms of dementia [55].

Taking into account all these evidence, we set out to investigate the effects of some nutrients on cognition, since the link between gut and brain (the gut-brain axis) is very close and begins *in utero*, moreover the Central Nervous System (CNS) and the Enteric Nervous System (ENS) originate from the same tissues during the embryonic development [11],[56]. In fact, it is known that human intestinal microbes regulate the elements of the gut-brain axis through immunological, endocrine and neuronal mechanisms [57],[58]. Gastrointestinal disorders, and the consequent gut-brain axis dysregulation, can lead to neurodegenerative diseases [59]. The variety of the microbiota plays a fundamental role in maintaining intestinal permeability, in order to avoid the absorption of toxic substances and the release of pro-inflammatory cytokines (i.e. the tumor necrosis factor-α and the interleukin-6) that may reach the brain and promote neuroinflammation associated with neurodegeneration [60]–[62].

However, while cellular and molecular mechanisms underlying the interaction between nutrition and its effects on brain functions have been widely investigated [63]–[66], we still know little about the epigenetic mechanisms behind these phenomena. For this reason, we have decided to investigate these mechanisms since they affect cellular and molecular phenomena governing the link between nutrition and cognitive functioning. Furthermore, a healthy diet can modulate epigenetic phenomena, opening up the possibility of preventing or modulating cognitive impairments.

Specifically, the term “epigenetics” refers to all those mechanisms regulating gene expression, maintaining the nucleotide sequence of DNA unchanged. During human normal aging, an unhealthy nutrition, together with a sedentary lifestyle, may cause deleterious epigenetic modifications during the whole life, inducing the expression of specific genes [67], which could strongly influence the metabolism and brain homeostasis.

The goal of this review is to describe how nutrition may affect cognitive functions over time through epigenetic mechanisms. In particular, we are interested in illustrating the main diet-dependent epigenetic phenomena (Table 1) since they play an essential role in the regulation of both physiological and pathological processes and in the neuronal reorganization or restructuring processes, including brain plasticity [68].

Therefore, we investigate the relationship between the main diet-dependent epigenetic mechanisms, such as DNA methylation and post-translational modifications of histone proteins, and cognition. The topics are discussed in both humans and animal models, from embryonic development to adulthood (Table 2). In particular, we focus on these processes as potential targets to analyze how some forms of cognitive decline could be prevented or delayed.

**Table 1.** Schematic illustration of the main diet-dependent epigenetic mechanisms reported in the text.

Epigenetic mechanism	Enzymes/processes involved	Effects on gene expression
DNA methylation The donation of a methyl group from a S-adenosyl-methionine (SAM) molecule to the position 5′ of the cytosine in CpG dinucleotide.	DNA methyl-transferases (DNMTs): DNMT1: maintenance of the methylation pattern after DNA replication. DNMT3a, DNMT3b: de novo methylation.	In the promoter of target genes: Hypermethylation: repression of gene expression. Hypomethylation: activation of gene expression.
Histone modifications The transfer of acetyl or methyl groups to lysine (Lys) and arginine (Arg) residues of histone proteins.	Histone acetyl-transferases (HATs): acetylation on Lys residues. Histone methyl-transferares (HMTs): methylation on Lys/Arg residues.	Histone acetylation: activation of gene expression. Histone methylation: activation/repression of gene expression.

**Table 2.** Illustration of some *dietary habits* investigated in the manuscript and their effects on cognitive functioning in both human and animal models via epigenetic mechanisms.

	Model	Eating habits	Epigenetic mechanisms	Health effects	References
Pregnancy and breastfeeding	Human	Maternal and/or paternal poor nutrition in the peri-conceptional period	Decreased fetal DNA methylation, both globally and at specific loci, such as IGF-2	Effects on fetal neurodevelopment and cognitive functioning later in life	[100], [101], [106]
		Folate intake from maternal diet	Regulation of DNA methylation through the folate-mediated one-carbon metabolism processes	Neural tube formation in the fetus, preventing spina bifida	[4], [116], [117]
		Maternal diet during the pregnancy and offspring's nutrition during the first two years of life	Several epigenetic mechanisms, mostly DNA methylation and histone modifications	Role in the risk of developing metabolic, cardiovascular and neurological diseases in the offspring	[62], [111]
		Alcohol abuse during pregnancy	Changes in the fetal epigenome	Increased risk for the fetus of developing fetal alcoholic syndrome (FAS) and fetal alcohol spectrum disorders (FASD)	[119], [120], [121], [125]
		LCPUFAs, cholesterol and carbohydrates of breast milk	Activation in the child of enzymes involved in histone post-translational modifications	Immune system strengthening, synaptic plasticity, neurogenesis, neurotransmission; lower risk of developing pediatric obesity, reducing inflammation and neuroinflammation later in life	[62], [140], [141], [142], [143], [147], [148]
	Animal models	Prenatal and postnatal alcohol exposure	Alterations in both global and specific (MeCP2 locus) DNA methylation and histone modifications	Developmental delay of the hippocampal dentate gyrus; alterations in the offspring's cognitive functions	[126], [127], [131]
		Fetal iron and zinc deficiency in rats	Alterations in histone modifications and DNA methylation in Bdnf gene	Effects on hippocampus	[132], [133], [134], [135]
		Deficiency of vitamin A during pregnancy in rats	Alterations in histone acetylation mediated by RAR-α	Risk of learning and memory impairments in the offspring	[136]
Adulthood and aging	Human	Dietary fibers deriving from vegetables consumption	Histone post-translational modifications	SCFAs produced by fibers fermentation maintain the gut integrity, preventing pro-inflammatory cytochines release and neuroinflammation	[62], [149], [150], [151], [152], [153], [154]
		Vitamin D	Not clear	Elevated serum are positively associated with attention and working memory, while a deficiency correlates with cognitive decline and executive functions impairments	[156], [157], [158], [159]
		Omega-3	Not clear	Role in the neuronal cell membrane fluidity, speed of signal transduction, neurotransmission, reduction of pro-inflammatory cytokines, neuroprotection against AD	[143], [160], [161]
		Deficiency in vitamins B6, B12 and folate	SAH accumulation and hyper-homocysteinemia with alterations in DNA methylation (e.g hypo-methylation in the promoter of PSEN1 gene)	Effects on cognition with loss of total brain volume and cognitive and memory decline; increasing amyloid processing, senile plaques deposition and cognitive impairment	[46], [47], [51]
		Vitamins A, E and C	Histone acetylation	Protective effects against oxidative stress and association with a decreased risk of developing AD	[177]
		Curcumin and EGCG		With their antioxidant properties, they participate in reprogramming neurogenesis from neural stem cells and reduce some forms of age-related cognitive dysfunctions	[137], [182]
	Other models (animal and cell models)	Folate and vitamin B12 deficiency in human neuroblastoma	DNA hypo-methylation in the promoters of PSEN1 and BACE1 genes	Increased gene expression with a higher production of beta-amyloid	[170]
		EGCG and curcumin in AD transgenic mice	Not clear	Reduction of inflammation and oxidative stress in the brain and in the levels of soluble beta-amyloid plaques	[168]
		Deficiency in vitamins B6, B12 and folate	SAH accumulation and hyper-homocysteinemia with alterations in DNA methylation	Effects on cognition with loss of total brain volume and cognitive and memory decline; increased amyloid processing and senile plaques deposition	[173], [174]
		LCPUFAs in neuroblastoma cells	Changes in histone methylation and acetylation	Reduced apoptosis	[180]

Note: AD: Alzheimer's disease; BACE1: beta-secretase-1; BDNF: brain-derived neurotrophic factor; EGCG: epigallocatechin gallate; FAS: fetal alcoholic syndrome; FASD: fetal alcohol spectrum disorders; IGF-2: Insulin-like Growth Factor-2; LCPUFAs: long-chain polyunsaturated fatty acids; MeCP2: methyl-CpG-binding protein; PSEN1: presenilin-1; RAR-α: retinoic acid receptor-α; SAH: S-adenosyl-homocysteine.

## 2. Methods

This review article investigates the relationship between the main diet-dependent epigenetic mechanisms and cognitive functioning, in humans and other mammals, emphasizing eating habits as strategies to prevent or modulate cognitive decline since very early stages of life.

**Figure 1.**
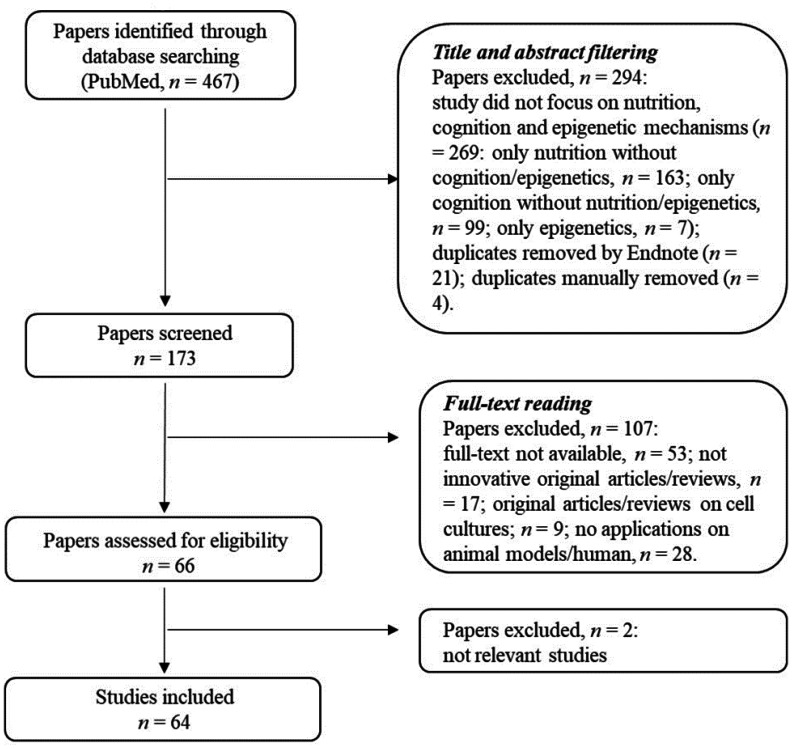
Adopted research methodology. The flow chart illustrates the steps of the selection procedure.

In this section, we focus on the definition of the research criteria and the strategy adopted to select the relevant literature. A flow chart with the selection procedure is reported in the Figure 1 and explained below.

### 2.1. Keywords definition

In this subsection we provide a brief definition of the main keywords used in the bibliographic research in order to clarify our inclusion and exclusion criteria.

Nutrition: all those manuscripts which take into account the consequences of eating habits on health.

Cognition: we consider studies examining cognitive abilities (i.e. attention, memory, learning) in physiological and pathological conditions and the underlying biological mechanisms.

Epigenetics: all those environment-dependent mechanisms which tend to regulate gene expression, keeping the nucleotide sequence of the DNA unchanged.

Environment: we selected all those papers that take into account factors such as smoking, alcohol consumption, stress, exposure to pollutants, and all eating habits, which can negatively influence human health by acting, among others, on the epigenetic mechanisms.

Pregnancy and breastfeeding: these are two crucial stages of life that we explicitly take into consideration to evaluate the effects of maternal (and paternal) eating habits on the offspring.

Neurodegeneration: we select all those papers describing how this condition could be improved from the early stages of life.

### 2.2. Literature research strategy

The aim of the manuscript is to describe how nutrition may affect cognitive functions through epigenetic mechanisms in human and other mammals, from pregnancy and breastfeeding to adulthood and aging. The topic is framed as a therapeutic target to analyze how certain forms of cognitive decline could be prevented or modulated since the early stages of life.

**Figure 2.**
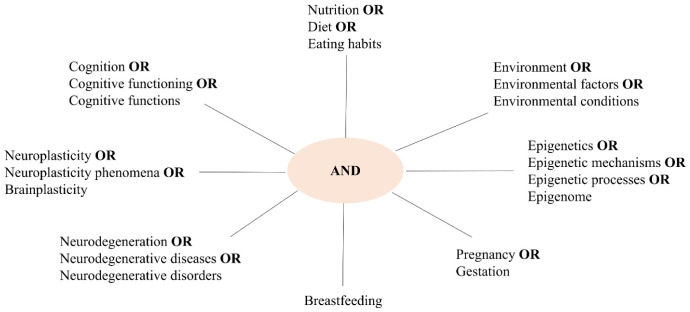
Scheme of the adopted search strategy for the papers selection.

For this purpose, a bibliographic research was performed in the PubMed database selecting the most interesting and relevant articles. In order to perform the search, the set of the keywords introduced above are applied as illustrated in Figure 2. The figure is composed of peripheral branches where each word is linked to its synonym by OR, and these branches converge in a central point (AND). By doing so, the research is carried out associating a word of a peripheral block with a word of another block.

### 2.3. Selection process

After a preliminary search, we identified 467 papers. In the first step, which involves reading the title and abstract of each article, we excluded the following manuscripts (n = 294):

All the manuscripts that do not focus on nutrition as main environmental factor;
Papers in which epigenetic mechanisms are not evaluated;
Studies where cognitive aspects are not evaluated.

In the second step of the bibliographic search, based on full-text reading, we adopted the following inclusion criteria:

All the papers that focus on eating habits, epigenetic mechanisms and cognition, analyzing these aspects in different stages of life (from embryonic development to adulthood and aging);
Innovative original articles/review articles;
Manuscripts describing studies on human and/or animal models;
All studies with available full-text.

In this phase of the selection process, the manuscripts were analyzed, leading to selection of 64 studies that are analyzed in this review.

## 3. The effects of nutrition on cognition during the lifetime: an overview on epigenetic mechanisms

The scarcity of resources has contributed to select an evolutionarily advantageous genotype over time, often referred to as the “thrifty genotype”. Such genotype favors nutrients absorption during periods of food availability. In fact, the tendency to accumulate calories as fat storage is likely adaptive during periods of scarce resources [69]. Furthermore, there was a direct relationship between the access to food and brain size, and even minor differences in the diet could have major effects on survival and reproductive success [70]. During human evolution, the development of activities such as cooking, foraging, walking or running was associated with the increasing size of the humanoid brain [71].

The “thrifty genotype” can be already activated in the fetus during pregnancy [72], since the mother's diet plays an essential role in the regulation of the epigenetic processes that might predispose the fetus to several diseases in adulthood. This phenomenon of metabolic memory, defined as the “Barker hypothesis”, shows that the *in utero* environment and the early postnatal life may correlate with long-term health outcomes [73].

This hypothesis has been accompanied over the years by the “thrifty phenotype” hypothesis [74], which states that malnutrition *in utero* would prepare the fetus to adapt to a potentially adverse environment after birth. In fact, malnutrition generates changes in the glucose-insulin metabolism [75], and prepares the fetus for early survival in nutritionally hostile environments. However, a subsequent rapid rate of growth after birth may predispose the individual to the risk of developing type 2 diabetes mellitus and metabolic syndrome later in life due to alterations in insulin secretion and insulin resistance [72],[76].

In support of this, it was seen that in developing countries and in rural areas food shortages and malnutrition contribute to the generation of “small-size” mothers resulting in uterine constriction of the fetus, probably mediated by the interaction between insulin-like growth factors and their binding proteins [77]. Caloric restrictions *in utero* and in early life represent a form of adaptation to a condition of food shortage; if these environmental conditions are present later in life, they represent an evolutionary advantage, resulting in cardiovascular health and metabolic improvement [78]. Conversely, individuals who have experienced fetal and early-life nutritional deficiencies, but then move to urban areas with high-caloric intake, are more predisposed to the development of cardiovascular diseases and metabolic syndrome [79].

The current widespread food availability, along with a sedentary lifestyle, turned the thrifty genotype and phenotype into a disadvantage, contributing to the onset of obesity, type 2 diabetes mellitus, cardiovascular disease, and their neurological complications [69],[80]. In particular, mid-life obesity is a risk factor for developing AD or vascular dementia during old age [81],[82] because of the comorbidities associated with obesity, such as type 2 diabetes mellitus, hypertension, hypercholesterolemia and insulin resistance [83]. Furthermore, an elevated body mass index (BMI) is associated with grey matter atrophy in several brain regions [84], and with reduced white matter integrity [85]. Together with morphological changes in the brain, neuroimaging studies demonstrated a relationship between obesity and functional brain alterations [86]–[88].

### 3.1. Nutrition and cognition in pregnancy and breastfeeding

In mammals, the first epigenetic processes underlying the regulation of gene expression occur in the early stages of the development, particularly during gametogenesis and in the pre-implantation embryo. In fact, after fertilization, the paternal genome in the zygote undergoes a demethylation process, while the maternal one is gradually demethylated during the blastocyst development. This loss of epigenetic information is necessary to create the new genome in the offspring. However, in order to reprogram the new methylome, some original information from the gametes must be maintained and are “imprinted” onto the new genome [4].

While it is widely known that maternal lifestyle in the peri-conceptional period and during pregnancy may affect the offspring, paternal contribution is often overlooked [89].

Several studies on animal models show that obesity and pre-conceptional exposure to pollutants, heavy metals or stress conditions affect the development of male germ cells through epigenetic changes affecting the offspring [90]. Based on this knowledge, the concept of “Paternal Origins of Health and Disease (POHaD)” was introduced as an extension of the “Developmental Origin of Health and Disease (DOHaD)” paradigm [89].

Few studies explore the epigenetic effects of the environment on the spermatozoa, taking into account some toxic agents or habits, such as pollutants [91], chemotherapy drugs [92], obesity and bariatric interventions [93],[94], smoking [95] and alcohol [96], which would be able to cause epimutations in male germ cells altering the global DNA methylation and histone post-translational modifications. To refer to these environmental factors, the “paternal exposome” expression has been used [91].

#### 3.1.1. Epigenetic mechanisms occurring during peri-conceptional period and pregnancy

DNA methylation is a process catalyzed by the DNA methyl-transferases DNMT1, DNMT3a and DNMT3b and it is reversible in Eukaryotes [97]. This involves the donation of a methyl group from a S-adenosyl-methionine (SAM) molecule to the position 5′ of the nucleotide cytosine in the CpG dinucleotide. In the human genome, the CpG dinucleotides are generally grouped in a very conserved regions called “CpG islands”, at least 200 base pairs long, with a C + G content higher than 50%.

DNA methylation occurs several days after fertilization, during the embryo implantation, through the DNA methyl-transferase DNMT1, and it is maintained through cell division. In fact, during DNA replication, the DNMT1 reads the methylated parental strand and methylates the newly synthesized complementary strand on the unmethylated CpG dinucleotide [98].

Environmental factors, such as toxic substances (heavy metals, volatile organic compounds, etc.), smoking, stress and nutrition [99] can influence these processes.

Human studies suggest that already in the peri-conceptional period both maternal and paternal poor nutrition can increase the risk of metabolic syndrome in the offspring through persistent changes in the DNA methylation of the fetus, which may last into adulthood [100]. These changes may occur both globally and at specific loci such as the Insulin-like Growth Factor-2 (*IGF-2*) gene, where a decrease in DNA methylation is associated with the risk of obesity, dyslipidemia and insulin resistance in adulthood [101]. Furthermore, global and gene-specific DNA hypo-methylation in the fetal brain, and the consequent gene expression alteration, may lead to behavior impairments [102]. Therefore, maintaining a healthy and balanced diet by both parents may reduce these risks [103].

It is widely known that during the gestation period, the mother's diet and habits play an essential role in the regulation of gene expression in the fetus, especially in the early months of development.

These phenomena are mainly due to the placenta which plays a critical role in the exchange of nutrients, oxygen and waste material between mother and fetus, and responds to maternal stimuli and perturbations with modifications in epigenetic mechanisms and gene expression [104],[105]. These epigenetic changes may have long-term effects on the fetal neurodevelopment [106].

Taken together, this data suggest that the crucial period for the establishment and maintenance of epigenetic changes in the offspring happens in the very early stages of the mammalian development [107], and this is confirmed by studies on mouse models [108],[109].

A valuable and rare example in support of these observations in humans is represented by individuals who were prenatally exposed to famine during the Dutch Hunger Winter in 1944–1945, compared with their unexposed, same-sex siblings [110]. This comparison shows that peri-conceptional exposure to famine is associated with decreased methylation in *IGF2* gene of 60 individuals 6 decades later, demonstrating how environmental conditions early in human gestation can lead to long-lasting changes in the epigenome.

Conversely, selecting individuals exposed to famine late in the gestation and born in or shortly after the famine, no differences are found with their unexposed siblings [100], suggesting that early stages of pregnancy represent a critical time window for the establishment of the epigenome.

It is also known that immediately after birth and in the early growth stages the environmental conditions either allow an adaptive response or predispose to several diseases in adulthood (see the above mentioned “thrifty phenotype” hypothesis, [72],[74]).

**Figure 3.**
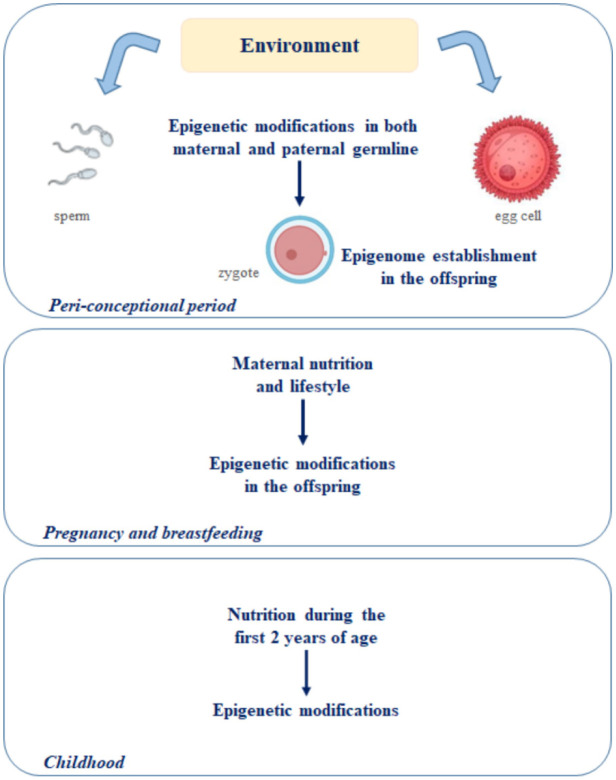
Schematization of the “first 1000 days of life” theory [62]. According to this theory, the period from the first day of pregnancy to two years of age is crucial for the establishment of the epigenome in the offspring.

Recent evidence converges on the fact that the crucial period for the later development would correspond to the first 1000 days of life [111]. According to the “first thousand days of life” theory, the period from the first day of pregnancy to two years of age is critical for the prevention of future diseases (Figure 3) [62]. In fact, the predisposition to cardiovascular, metabolic and neurological disorders in the offspring begins *in utero* and is associated with the inheritance of epigenetic alterations. As seen before, these depend on the maternal nutrition and lifestyle during the pregnancy and on the offspring's nutrition during the first two years of life [112],[113]. Therefore, it is possible to hypothesize a connection between the 1000 days of plasticity and the predisposition to neurodegeneration later in life [62].

Furthermore, the strong association between maternal nutritional status and the risk of developing metabolic, cardiovascular and neurological diseases in the offspring [114], passing through epigenetic alterations [115], is emphasized by some papers in which micronutrients are examined. For example, some methyl donor micronutrients, such as folate, methionine, choline and betaine play a key role in the regulation of DNA methylation through the folate-mediated one-carbon metabolism processes [116],[117]. In particular folate, which is present in some green leafy vegetables and whose supplementation is recommended to pregnant women, has a fundamental role in the correct neural tube formation in the fetus, preventing its defects (spina bifida) [4].

Some preliminary studies on alcohol consumption in pregnant or lactating Italian women reveal that this habit is not negligible [118]. The first studies on the harmful effects of alcohol in pregnancy date back to 1960s. Alcohol abuse during pregnancy increases the risk for the fetus of developing fetal alcoholic syndrome (FAS), which is characterized by facial abnormalities, growth retardation and brain damage (intellectual difficulties and behavioral disturbances) [119], and fetal alcohol spectrum disorders (FASD), which is a neurodevelopmental deficit characterized by hyperactivity, attention deficit and cognitive disability [120],[121]. The pathogenetic mechanisms behind these clinical conditions are not clear, but the direct toxic effect of ethanol, the cytotoxicity induced by alcohol catabolism, the production of oxygen free radicals and the inhibition of cell adhesion molecules may have a role in the pathology [122]–[124]. Furthermore, some evidences suggest that gestational alcohol exposure may result in lasting changes in the fetal epigenome [125].

A study on FASD animal models show that prenatal and postnatal alcohol exposure increases the activity of the DNMTs in the fetus, modifying the expression of the methyl-CpG-binding protein (*MeCP2*) gene, which is essential for the normal functioning of nerve cells [126]. The hippocampus of these fetuses exhibits developmental delay with fewer cells in the dentate gyrus [127]. In addition to this, exposure to alcohol during gestation affects the neurulation process, altering DNA methylation and the expression of several genes, including those implicated in cell cycle, growth and apoptosis, modulating the development of the fetus at early stages [128].

Histone post-translational modifications are also part of the epigenetic effects derived from alcohol consumption in pregnancy, but the exposure time and the different responsiveness of brain areas contribute to the inconsistent results [129].

As seen before, eating habits may also affect the epigenome of the paternal germline, influencing the offspring's health already in pre- and peri-conceptional periods [89],[90].

Surprisingly, paternal alcohol consumption can also be a risk to the offspring. In fact, studies in both animal models and humans demonstrate how ethanol is able to alter DNA methylation and histone post-translational modifications in both testis and developing sperm cells [4],[96],[130]. How these epigenetic changes may affect embryonic development and cognitive functions is still unclear, but a recent study show altered gene expression in the offspring neocortex and intraneocortical connections. Both male and female offspring suffer alterations in cognitive functions, such as sensorimotor integration, balance, coordination and short-term motor learning [131].

Histone methylation is a process catalyzed by the histone methyl-transferases (HMTs), that transfer one to three methyl groups from SAM to lysine and arginine residues of histone proteins (H2A, H2B, H3 and H4), which play an essential role in the process of chromatin compaction. The methylation is associated with activation/repression of the gene expression depending on the position on which it occurs. In particular, dimethylation and trimethylation on lysine 4 of histone H3 (H3K4me2 and H3K4me3) seem to be a global epigenetic marker in euchromatic regions, while methylation on lysine 9 of histone H3 (H3K9me) is an epigenetic marker crucial for the heterochromatin formation and transcriptional silencing. Otherwise, the acetylation of lysine residues on the N-terminal tails of histones is mediated by the histone acetyl-transferases (HATs) and it is always associated with increased gene expression, favouring the chromatin opening [68].

Among micronutrients, zinc and iron are involved in the regulation of some epigenetic mechanisms in response to the maternal diet during pregnancy in murine models [132]. In particular, they play a role in both histone modifications and DNA methylation [133],[134]. Fetal iron deficiency in rats leads to alterations in histone methylation and acetylation and in DNA methylation in the *Bdnf* gene, resulting in lower transcription in hippocampus. The effects of these processes are also observed in adulthood [135].

During pregnancy and in the first years of life, vitamin A plays an important role in the development of learning and memory processes of the fetus, so its deficiency (VAD) adversely affects the development of the nervous system. In the hippocampus, the nuclear receptor for retinoic acid-α (RAR-α) is one of the most important biomarkers from this point of view, and its interaction with the histone acetyltransferase CREB-binding protein (CBP) is examined in 8-week-old VAD rats, where lower levels of histone acetylation are observed, due to a dysregulation in histone acetylation mediated by RAR-α. Since histone acetylation is involved in the development of the nervous system, learning, memory and the pathogenesis of neurodegenerative diseases induced by vitamin A deficiency, these results support the importance of vitamin A assumption during pregnancy and early life in order to prevent impairments of learning and memory in adulthood [136].

Furthermore, the antioxidant properties of vitamin A are well known, and they might prevent damages to histone acetylation mechanisms induced by reactive oxygen species [137].

#### 3.1.2. Epigenetic mechanisms during breastfeeding

Breast milk, compared to formula milk, has lower protein content, but contains biologically active compounds [138], and higher levels of long chain polyunsaturated fatty acids (LCPUFA), cholesterol and non-digestible carbohydrates, which act as a substrate for the beneficial bacterial strains that grow in the gut microbiota (*Bifidobacterium*, *Lactobacillus*) and contribute to the strengthening of the immune system [139]. In fact, the short-chain fatty acids produced by the intestinal bacteria from the dietary fiber fermentation influence the activity of the enzymes involved in the post-translational modifications of the histones and thus gene expression. This would seem the main reason why breastfeeding contributes to the immune system strengthening of the baby during the first 6 months of life [140]. The n-3 and n-6 LCPUFAs are essential for brain development and cognitive functions of the child. In fact, they contribute to myelin sheath formation and enhance synaptic plasticity [141], and are involved in several processes ranging from cell membrane fluidity to gene expression regulation [142]. In particular, DHA plays a role in neurogenesis, neurotransmission and protection against oxidative stress [143], and its accumulation starts *in utero* in correspondence with the development of the gray matter of the fetus [144],[145]. Furthermore, breastfed newborns show higher levels of DHA in erythrocytes than formula-fed infants [146], demonstrating that the mother's diet affects the offspring before birth and in the early stages of life [141]. Ultimately, n-3 LCPUFAs uptake from the mother's diet during pregnancy and breastfeeding has long-term effects on visual acuity, psychomotor development and mental skills in children [141].

Breast milk is known to reduce the risk of pediatric obesity [147],[148]. Since obesity is linked to inflammation and to neuroinflammation, one could speculate that the risk of developing neurodegenerative diseases in adulthood might be reduced by breastfeeding [62].

The benefits of breast milk involve epigenetic mechanisms that are not yet entirely clear, but which may influence gene expression of the offspring with both short-term and long-term effects.

### 3.2. Nutrition and cognition in adulthood and aging

As mentioned above, there are several nutrients that contribute to cognitive development across the lifespan. This is due to the close communication between the gut microbiota and the brain [149] and to the role of some nutrients along the gut-brain axis. For example, the short-chain fatty acids (SCFAs) propionate, butyrate and acetate are produced by the fermentation of dietary fibers derived from vegetables consumption, and their beneficial effects in preventing cardiovascular disease, metabolic syndrome, cancer and neurodegenerative diseases are known [150]. SCFAs act as both signal transduction molecules through G protein-coupled receptors and regulators of gene expression through histone post-translational modifications, and exert their action on the brain through immune, endocrine, vagal and other humoral pathways, since their ability to cross the blood-brain barrier [151].

**Figure 4.**
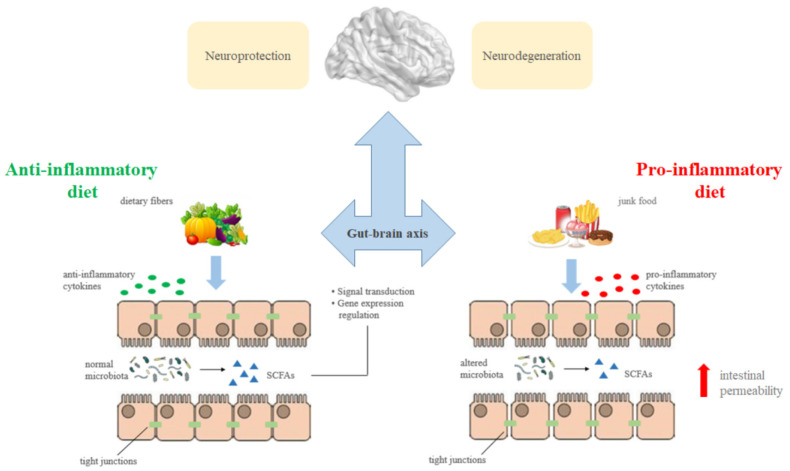
Effects of nutrition on cognitive functioning through the gut-brain axis. Short-chain fatty acids (SCFAs) are produced by the microbial fermentation of dietary fibers deriving from vegetables consumption and act as both signal transduction molecules through G protein-coupled receptors and regulators of gene expression through histone post-translational modifications. They play a key role in preventing neurodegenerative diseases due to their ability to cross the blood-brain barrier and to exert their action on the brain through immune, endocrine, vagal and other humoral pathways, preventing the increase in intestinal permeability and the release of pro-inflammatory cytokines usually associated with a poor nutrition.

They play an essential role in maintaining microbiota diversity and in regulating intestinal permeability [152]–[154]. In fact, the alteration of intestinal microbes involves an increase in the gut permeability with the consequent passage of toxic substances and pro-inflammatory cytokines (Figure 4), thus feeding inflammatory and neuroinflammatory processes [62],[151].

Among micronutrients, vitamin D promotes intestinal absorption of minerals such as calcium, magnesium and phosphorus [155]. Its metabolites and receptors (VDR) have been found in the brain [156],[157], particularly in neurons and glial cells of the hippocampus, hypothalamus, cortex and subcortex [157]. Elevated serum levels of vitamin D are positively associated with attention and working memory [158], while vitamin D deficiency correlates with cognitive decline [54] and executive functions impairments [159].

Likewise, omega-3 fatty acids contribute to the development of the brain and its functions [143]. In particular, DHA plays a role in the neuronal cell membrane fluidity, affects the speed of signal transduction, neurotransmission and formation of lipid rafts [160],[161]. Furthermore, it is involved in gene expression regulation, ionic channels activity, and it is metabolized into neuroprotective metabolites [29],[162],[163].

The factors that contribute to the onset of the most common neurodegenerative diseases, such as late onset AD (LOAD), are numerous and include the interaction between genetic factors and environmental conditions.

Interesting studies on monozygotic twins confirm this and show the influence of the environment in determining the age of onset of the disease, which may differ in the 4–16 years range among monozygotic twins [164]. According to these studies, among the environmental factors associated with LOAD there are exposure to heavy metals, electromagnetic fields, chemicals, depression, hypertension, stroke, diabetes, high cholesterol levels, obesity, brain trauma, smoking and stress. However, among the protective factors there are physical activity [165], mental activity [166], education, social relations and a balanced nutrition [164].

For example, it is known that the consumption of fish, which is rich in omega-3, reduces the risk of developing AD, while antioxidants such as vitamin E and plants polyphenols curcumin and green tea epigallocatechin gallate (EGCG) have neuroprotective activity which may have an important role in preventing AD [167]. In fact, DHA and eicosapentaenoic acid (EPA) have beneficial effects due to the reduction of pro-inflammatory cytokines and of obesity-induced insulin resistance.

In AD transgenic mice exposed to curcumin or EGCG, a significant reduction of inflammation and oxidative stress in the brain and a reduction in the levels of soluble beta-amyloid plaques are observed [168]. Furthermore, an interesting study on the brain of AD rat models demonstrates that there is a link between the deficit in cerebral energy metabolism and the dysfunction of the cholinergic system, providing a useful tool to study the complex mechanisms behind two interrelated phenomena characterizing AD from the early stages [169].

*In vitro* studies on human neuroblastoma show a DNA hypo-methylation in the promoters of *PSEN1* and *BACE1* genes in conditions of folate and vitamin B12 deficiency, resulting in increased gene expression associated with an increased production of beta-amyloid. In this case, the addition of S-adenosyl-methionine restores *PSEN1* and *BACE1* expression to baseline levels [170].

Although not fully clear, some evidence shows that the activation of epigenetic mechanisms could be behind these processes. In fact, global changes in the DNA methylation and histone modifications are observed in the brains of AD cases compared to controls, such as DNA hypo-methylation in the entorhinal cortex [171] of AD patients.

#### 3.2.1. DNA methylation

DNA methylation, as we have seen, is mediated by the DNMTs and the methyl donor SAM, which is an intermediate of the homocysteine cycle. This process is regulated by vitamins B6, B12 and folate, and its alteration leads to aberrant DNA methylation related to several neurological disorders and autoimmune diseases. Several cases of neurodegenerative diseases such as AD, PD, Amyotrophic Lateral Sclerosis (ALS) and Frontotemporal Dementia (FTD), result from a combination of genetic and environmental factors, which is not yet well defined. An unbalanced intake of nutrients, together with epigenetic phenomena, plays an important role in the onset and progression of these disorders [172].

As mentioned previously, SAM is the donor of methyl groups used to methylate DNA, RNA, proteins and lipids, and it is an intermediate of homocysteine cycle, governed by vitamins B.

**Figure 5.**
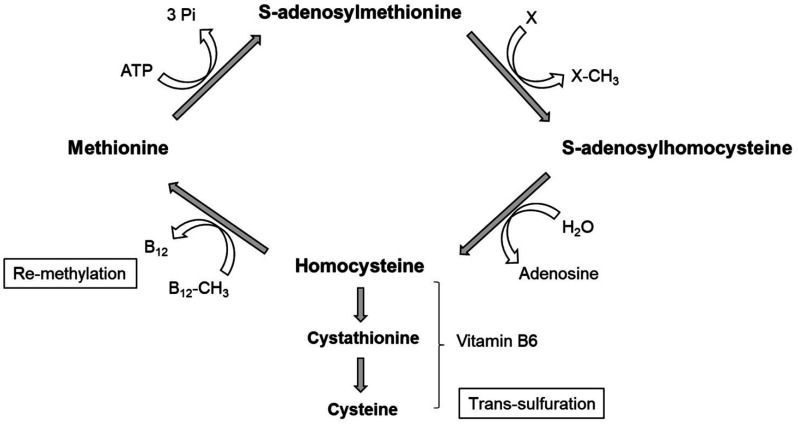
The methyl cycle. After the methyl group donation to a precursor molecule (X: DNA, RNA, proteins, lipids), S-adenosylmethionine (SAM) becomes S-adenosylhomocysteine (SAH), which is usually turned into homocysteine (HCY). HCY can undergo two fates: in the re-methylation pathway, it is re-methylated and converted into methionine (precursor to SAM) in a reaction involving vitamin B12; in the trans-sulfuration pathway, HCY is first converted to cystathionine and then to cysteine in a reaction that involves vitamin B6.

After the methyl group donation, SAM becomes SAH (S-adenosyl-homocysteine), which is usually turned into HCY (homocysteine). Homocysteine can either go in the re-methylation pathway, where HCY is re-methylated and converted into methionine (precursor to SAM) in a reaction involving B12 and folate; or go in the trans-sulfuration pathway, where HCY is first converted to cystathionine and then to cysteine in a reaction involving vitamin B6 (Figure 5) [172].

The alteration of this cycle due to vitamins B deficiency involves SAH accumulation, resulting in hyper-homocysteinemia, and the decrease in the SAM/SAH ratio, altering the activity of the methyl-transferases. This mechanism may be the way by which vitamins B deficiency is linked to neurodegeneration [173].

It is seen that vitamin B12 deficiency and hyper-homocysteinemia may affect cognition with loss of total brain volume and cognitive and memory decline [46],[47], through pathways involving methylation, redox potentials with altered calcium influx, tau protein and beta-amyloid accumulation, apoptosis and neuronal death [51].

In fact, in AD vitamins B deficiency is associated with decreased SAM/SAH ratio, with inhibition of DNA methyl-transferases activity and stimulation of the DNA demethylases activity. The activity of the demethylases would involve a hypo-methylation in the *PSEN1* gene promoter, resulting in the gene overexpression, increasing in the amyloid processing, senile plaques deposition and cognitive impairment [174]. Several studies support this theory, while others claim that the alteration of DNA methylation could be regulated by amyloid rather than be regulating the amyloid processing.

Studies conducted on cell cultures and animal models suggest that the alteration of DNA methyl-transferases activity is related to neurodegeneration. Indeed, aberrant DNA methylation and neurodegeneration can be due to mutations in the genes encoding the DNA methyl-transferases. For example, mutations in the *DNMT1* gene, which cause a loss of function of the enzyme and abnormal methylation patterns, are associated with dementia, sensory neuropathy, hearing loss [175], cerebellar ataxia and narcolepsy [176]. In addition, overexpression of the *DNMT3a* gene induces neurodegeneration and apoptosis, while a depletion/loss of function mutation in the same gene reduces apoptosis in the motor neurons. Alterations in *DNMT1*, *DNMT3a* and in DNA methylation levels are found in the motor neurons in ALS patients [172].

#### 3.2.2. Histone modifications

As seen previously, in the homocysteine cycle, SAM is the donor of the methyl groups used to methylate the DNA, the RNA, proteins and lipids, in a process regulated by vitamins B. An alteration of this one-carbon metabolism leads to hyper-homocysteinemia with changes in methyl-transferases activity, and this affects both DNA and histone methylation.

Vitamins B deficiency leads to a hyper-homocysteinemia condition and is related to neurological issues, poor cognition and AD through these epigenetic mechanisms [177],[178]. Conversely, supplementations with vitamins B12, B6 and folate reduce serum concentrations of homocysteine, slowing down cognitive decline [179].

Furthermore, vitamins A, E and C have antioxidant properties, so they can reduce abnormal histone acetylation modifications due to an oxidative environment. Increased intake of these antioxidant molecules from food has protective effects against oxidative stress and is associated with a decreased risk of developing AD [177].

Several evidences support the role of PUFAs in slowing cognitive decline. A study on neuroblastoma cells demonstrates that DHA can reduce histone deacetylases (HDAC1, HDAC2 and HDAC3) activity resulting in global increased acetylation on H3K9 residues. In addition, DHA promotes histone demethylation processes. These histone modifications together lead to an active transcription of genes associated with reduced apoptosis [180]. In humans, a study on peripheral blood mononuclear cells of AD patients shows how these cells decrease their ability to produce specialized pro-resolving mediators over time with the disease progression. These mediators derive from n-3 and n-6 PUFAs with anti-inflammatory properties. n-3 fatty acids supplementation for 6 months prevents these mediators reduction, demonstrating the importance of the PUFAs in hindering age and AD-related deterioration [181].

Curcumin is a strong antioxidant with widely known beneficial effects for AD. It inhibits different isoforms of HDACs, participating in histone acetylation processes which result, among other things, in reprogramming neurogenesis from neural stem cells [137]. Similarly as curcumin, green tea EGCG has neuroprotective properties and its regular consumption can reduce some forms of age-related cognitive dysfunctions through its mechanisms of action, including inhibition of the HDACs [182].

## 4. Conclusions

Among environmental factors determining neuroplasticity phenomena, nutrition plays an essential role in inducing structural and functional changes in the brain, determining effects on cognition [8]–[10].

In this review, we investigated the main diet-dependent epigenetic mechanisms involved in cognitive functioning, from the embryonic development to adulthood, and analyzed them as potential therapeutic target for the prevention of cognitive impairments from the early stages of life.

In fact, as seen so far, epigenome alterations play a key role in the susceptibility to several diseases, including cognitive deterioration. Although the mechanisms underlying these processes are not fully understood, a healthy nutrition affects epigenetic processes, helping to lower the risk of developing neurological diseases from very early in life.

The link between nutrition and cognitive functioning derives from the close interconnection existing between the gut microbiota and the cognitive mechanisms passing through the gut-brain axis. In fact, an unhealthy diet may modify the gut microbiota, leading to an alteration of intestinal permeability, with consequent absorption of toxic substances and release of pro-inflammatory cytokines, which mediate inflammation and neuroinflammation [62].

As we have seen, the predisposition to cardiovascular, metabolic and neurological disorders in the offspring derives from diet- and lifestyle-dependent epigenetic changes occurring in both maternal and paternal germ lines during the peri-conceptional period [89],[90],[100]. Pregnancy and breastfeeding are also crucial for the establishment and maintenance of the epigenome in the offspring, demonstrating that there is a susceptibility time window to epigenetic modifications which may predispose the offspring to cognitive impairments in adulthood.

These evidences led researchers to formulate the “first thousand days of life” theory, indicating the period from the first day of pregnancy to two years of age as critical for the prevention of diseases [62]. Therefore, it is possible to hypothesize a connection between the 1000 days of plasticity window and the predisposition to neurodegeneration later in life [62].

Based on these evidences, one might suggest some behavioral measures to lower the risk of neurodegenerative disorders: 1) maternal and paternal lifestyles and nutrition should be healthy already during the peri-conceptional period; 2) during pregnancy and breastfeeding, the mother should adopt a healthy diet and lifestyle, avoiding harmful environmental conditions such as exposure to pollutants, smoking, stress or alcohol consumption; 3) breastfeeding should be promoted for its beneficial effects on brain development and cognitive functions of the child; 4) the first two years of age are a crucial time window for brain epigenetic susceptibility, then the mother should educate the child to a healthy nutrition and lifestyle from early childhood; 5) a good amount of fish and dietary fibers should be included in the diet due to their anti-inflammatory and neuroprotective properties both in early life and adulthood.
